# Omalizumab vs. placebo in the management of chronic idiopathic urticaria: a systematic review

**DOI:** 10.1186/s40413-014-0050-z

**Published:** 2014-12-31

**Authors:** Diana C Carrillo, Mario Sanchez Borges, Elizabeth García, Eduardo Egea, Carlos D Serrano

**Affiliations:** 1 Department of Internal Medicine, Fundación Valle del Lili, Carrera 98 # 18-49, Cali, Colombia; 2 Allergy and Clinical Immunology Department, Centro Médico-Docente La Trinidad, Avenida Intercomunal La Trinidad, El Hatillo, Apartado Postal 80474, Caracas, 1080-A Venezuela; 3 Allergy Unit, Fundación Santa Fé de Bogotá, Carrera 7B # 123-90, Bogotá, Colombia; 4 Medicine Department, Universidad del Norte, Kilometro 5 vía a Puerto Colombia, Barranquilla, Colombia; 5 Allergy Unit, Fundacion Valle del Lili, Carrera 98 # 18-49, Cali, Colombia

**Keywords:** Antibodies, Monoclonal, Humanized, Urticaria, Angioedema

## Abstract

**Objectives:**

To examine the evidence derived from randomized controlled clinical trials on the efficacy and safety of omalizumab compared to placebo in controlling symptoms of chronic idiopathic urticaria/chronic spontaneous urticaria (CIU/CSU).

**Data source:**

The electronic databases *PubMed, Medline, EMBASE, Biomed Central, The Cochrane Central Register of Controlled Trials (CENTRAL), Wiley, OVID,* and *HighwirePress* were reviewed. The date limit was set to May 31th, but it was extended to September 30th of 2014 due to a new publication. No language restriction was used. The articles included were randomized trials controlled with placebo in individuals older than 12 years diagnosed with CIU/CSU refractory to conventional treatment, the **intervention** being, omalizumab at different doses, and **the comparison**, placebo. The primary outcome was symptom improvement according to the weekly score of urticaria severity (UAS7), the itch severity score (ISS), the weekly score of number of urticarial lesions, the dermatology life quality index, and the chronic urticaria quality of life questionnaire (CU-QoL). Databases were searched using the following Mesh or EMTREE key words including as intervention “omalizumab” or “humanized monoclonal antibody,” compared to placebo and the disease of interest “urticaria” or “angioedema”. The title, abstract and article were reviewed by two independent investigators, according to the selection criteria in each of the databases. An assessment of the quality of the articles was performed according to the bias tool from the studies of the Cochrane Collaboration. Information such as author data, date of study, number of participants, interventions, dose and frequency of administration, comparison, time of follow-up, measurements of weekly score of urticaria activity, pruritus severity score, weekly urticarial lesions, percentage of angioedema and post-treatment change were extracted. Frequency of adverse events and the ones suspected to be caused by the intervention drug were included.

**Results:**

770 records were identified in all databases described. 720 were eliminated for failing to meet the inclusion criteria in the first review or for duplicate records. 24 articles were reviewed by abstract, 18 additional articles were further removed, leaving 6 records for inclusion. An experimental study was excluded because it wasn’t randomized. Five studies were finally included, with 1117 patients, of these 831 received a dose of omalizumab of 75 mg (183 patients, 16.38%), 150 mg (163 patients, 14.59%), 300 mg (437 patients, 39.12%) or 600 mg (21 patients, 1.8%), as a single dose, or every 4 weeks until 24 weeks maximum. The average age was 42.07 years, predominantly female gender and white ethnicity. It was observed that the use of omalizumab 300 mg lowered the weekly scores of urticarial activity in 19.9 vs. 6.9 on placebo (p <0.01), 19 vs 8.5 and 20.7 vs 8.01 in three studies, the weekly ISS (−9.2 vs. - 3.5, p <0.001, −9.8 vs −5.1 p < 0.01, −8.6 vs −4.0 and −9.4 vs −3.63 p <0.001 in four studies), and the percentage of angioedema-free days (omalizumab 95.5% vs. placebo 89.2% p <0.001, and 91.95% vs. 88.1% p <0.001 in two of the studies respectively).

**Limitations:**

The different doses used throughout the study, time of administration and follow-up periods ranged from single dose to monthly dose for 24 weeks. Therefore no meta-analysis of the review was conducted.

**Conclusions and implications of the main findings:**

Despite the limitations, it is considered that omalizumab 300 mg is effective in treating chronic idiopathic urticaria refractory to H1 antihistamines. Further studies are required to determine the duration of effective treatment.

**Registration number of the systematic review:**

http://www.crd.york.ac.uk/PROSPERO/display_record.asp?ID=CRD42014010029#.VJDr1vl5PpM (PROSPERO. International Prospective Register of Systematic Reviews).

**Electronic supplementary material:**

The online version of this article (doi:10.1186/s40413-014-0050-z) contains supplementary material, which is available to authorized users.

## Introduction

Chronic idiopathic urticaria/chronic spontaneous urticaria (CIU/CSU), is a skin disease characterized by recurrent appearance of wheals, angioedema, or both, occurring at least twice a week for more than 6 weeks [1]. Its course is self-limiting with spontaneous remissions and relapses [2]. It is an important cause of morbidity and even though it has a very low risk of endangering life, it has a high impact on quality of life [3],[4]. This disease affects at least 0.5-1.0% of the population and 40% may present urticarial lesions up to 10 years later [1],[4]-[7].

The pathogenesis of CIU/CSU is not completely understood. It is considered that mast cell degranulation and histamine release play a major role, however, in more than half of the patients there is no established triggering allergic event that can be made responsible for the mast cell activation and so it is called chronic idiopathic (or spontaneous) urticaria [3],[4]. In some cases the presence of immunoglobulin G antibodies to the high affinity receptor for IgE (FcεRI) alpha subunit or to IgE itself has been documented [2],[4].

Treatment options are few and usually off-label [3]. The EAACI/GA^2^LEN/EDF/WAO (European Academy of Allergology and Clinical Immunology, Global Allergy and Asthma European Network, European Dermatology Forum and World Allergy Organization, respectively) guidelines recommend the use of non-sedating antihistamines at conventional dose as a first line of treatment, the increase of their dosage up to fourfold as a second line, and the use of Omalizumab, montelukast, or systemic immunosuppressants as cyclosporine A for the third line of treatment, and corticosteroids for a short course for disease exacerbations [8].

For those patients refractory to standard medical management, the use of omalizumab, an IgG1k type monoclonal antibody that binds to free Immunoglobulin E in the blood, has been proposed as a treatment [3]. It also reduces the expression of Fc epsilon RI in circulating basophils [9]. The mechanisms by which it reduces the activity of urticaria are not precisely known, but it has been reported to diminish the expression and activation of mast cells of the skin, and the subsequent release of histamine and other mediators such as leukotrienes, tryptase, chymase, prostaglandin D2 and cytokines [9],[10].

Omalizumab is approved for the treatment of moderate to severe persistent asthma inadequately controlled with inhaled steroids and positive *in vivo* or *in vitro* tests for perennial aero-allergens and achieving improvement of up to 75% compared to baseline, and now is approved for patients with CIU/CSU [8 ]. Its main adverse effect is anaphylaxis, with a mean frequency of 0,14% in asthmatic patients that receive the drug [11]; however, side effects reported in patients with CIU/CSU occured in 1-10% and included local reactions at the injection site (swelling, redness and itching), sinusitis, headache, arthralgia, and upper respiratory infections, with lack of serious and severe adverse events probably due to the lower doses used for this indication [9],[12]. In some cases there has been a reaction similar to serum sickness. Injection is recommended to be applied at the hospital and followed by an observation period of two hours after the first dose and 30 minutes after subsequent injections.

The effect of omalizumab in CIU/CSU has been measured using the Urticaria Activity Score (UAS), which assesses the number of urticarial lesions and intensity of itching that occurs in one or 7 days, or the Dermatology Life Quality Index (DLQI) which measures the impact of skin diseases on quality of life, among others [10]. Given the above, we decided to conduct a systematic review of the literature where the efficacy of omalizumab is evaluated and compared to placebo, in patients older than 12 years with CIU/CSU in terms of symptom improvement.

## Methods

A systematic literature review was conducted to identify all studies evaluating the efficacy of omalizumab in the treatment of CIU/CSU. The study population included individuals older than 12 years diagnosed with CIU/CSU who had failed to treatment with H1 antihistamines. The intervention was the use of omalizumab at different doses (75 mg, 150 mg, 300 mg, 600 mg) subcutaneously every 4 weeks, and the comparison was to placebo. The main outcome measures were symptom control established by: 1) **weekly score of urticarial activity** (Urticaria Activity Score -UAS- 7), this being the sum of the individual scores of daily urticaria activity (UAS) in the last 7 days, it can vary from 0–42 points per week (0–6 days); 2) the **weekly Itch Severity Score** (Itch Severity Score-ISS-) consisting of average daily sum (morning and evening) of pruritus scores in the last 7 days, with 0 being no pruritus, 1 mild, 2 moderate, 3 Severe, with a value from 0 to 21; 3) **weekly number of** wheals measured twice daily (morning and evening) on a scale of 0 when there was no urticaria to 3 when there were over 12 urticaria lesions (0: none, 1: 1–6, 2: 7–12 3:> 12 lesions) with a weekly value from 0 to 21; 4) **largest wheal size** (0: none, 1: <1.25 cm, 2: 1.25 cm-2.5 cm, 3:> 2.5 cm) twice daily for a week; 5) **The Dermatology Life Quality Index**, which is a life quality scale that includes 10 items on 6 topics: symptoms and feelings, daily activities, leisure, work/school, personal relationships and treatment. Each item is scored on a 4-point scale ranging from 0 to 3. The overall score of DLQI ranges from 0–30 by adding each of the scores of each item. A high score indicates a big change in quality of life; 6) **the chronic urticaria quality of life questionnaire** (Chronic Urticaria Quality of LifeQuestionnaire-Cu-Q2oL-) which includes physical, emotional, social and practical domains that characterize this disease; and 7) **presence of angioedema** in proportion [13]-[15]. Safety events were also included such as 8) **frequency of adverse events**; 9) **serious adverse events**; and 10) **adverse events suspected to be caused by the study drug**.

Double-blind placebo-controlled randomized clinical trials (RCTs) were selected. A placebo-controlled randomized clinical trial was defined as a prospective study that included individuals randomly assigned to one or more alternatives including placebo.

A comprehensive and reproducible search for original work was performed in electronic databases related to health *PubMed, Medline, Cochrane Central Register of Controlled Trials (CENTRAL), Trip Database, Wiley, Biomed Central, Highwire Press, EBSCO and OVID* using MeSH terms, EMTREE terms or keywords that included “Omalizumab” or “Antibodies, Monoclonal, Humanized” or “placebo” and “urticaria” or “angioedema”. For the PubMed database we used (“Antibodies, Monoclonal, Humanized” [Majr]) OR “Placebos” [Mesh]) AND (“Urticaria” [Mesh] OR “Angioedema” [Mesh]) [16]. This data search was conducted without language filter and with a date limit to May 31th of 2014, but it was extended to September 30th of 2014 due to a new publication this month. The search was restricted to humans, placebo-controlled RCT. The placebo-controlled RCTs that evaluated the effect of omalizumab on controlling symptoms in patients with chronic idiopathic urticaria were eligible. Other sources of information such as conferences, conference proceedings, posters, newspapers and secondary sources such as systematic reviews, and gray literature were not included. A review by title was performed in the described databases and in those who generated uncertainty, the abstract or the article was revised. Studies unrelated to the question of interest in the title or abstract were excluded and the studies that met the requirements specified for the quality review were included. From the selected articles, we obtained by means of two independent reviewers using a format of data collection, the study reference, work date (year), authors, quality RCT (random sequence, allocation, blinding), eligibility criteria (population , intervention, comparison and outcome, reason for excluding), study design, study duration, number of participants in the intervention group, number of participants in the placebo group, age, sex, country, comorbidities, ethnicity, drug doses, days of treatment, route of administration, side effects, number of patients lost to follow up, outcomes as mean or median differences according to the difference between the baseline measurement and follow-up measurement or percentage change as reported, confidence intervals if included, subgroup analysis if applicable, sources of funding, if they had discrepancies between the two reviewers, a third reviewer assessed the article. A tool provided by the Cochrane Collaboration for assessing the domains of sequence generation, allocation, masking, selective results and other items was used [17]. A format in Excel 2013 for data collection was built and a narrative description of the studies and their characteristics was performed because the differences in interventions (differences in dose and frequency of administration) and in the outcomes were anticipated. Therefore no meta-analysis was considered for publication. The present study had funding from Novartis Pharmaceuticals.

## Results

**Figure 1:**
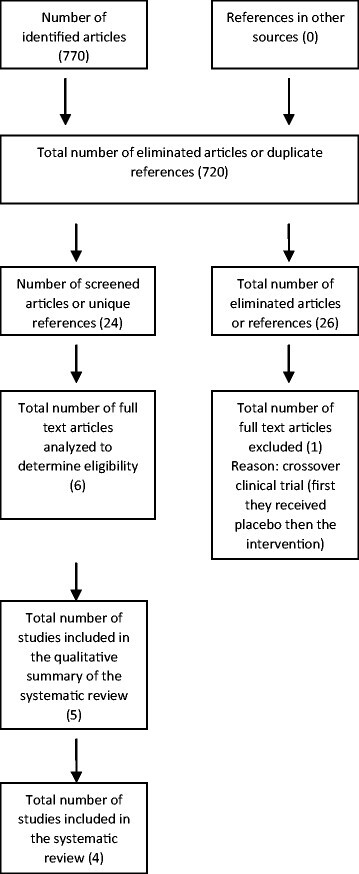
**Flowchart of identified, removed and included studies in the systematic review.**

770 records (Figure 1) were obtained, of these 720 results were excluded when reviewing the title and because of repeated citations, and 18 were excluded when reviewing the abstract. 6 articles were included for final review. Of these 1 was excluded for not meeting inclusion criteria (no randomization) [18]. There were 5 studies left to review quality criteria [19]-[23]. The assessment of the studies’ methodological quality is outlined in Table 1 [17]. In general, a low risk of bias was identified in the sequence of random generation among studies, allocation concealment and blinding. There were three studies where there was no blinding of the staff preparing the medication at the center; however, this was not the staff responsible for the administration of the drug [19]-[23]. In each study the percentage of patients lost was reported. The risk of bias due to patients lost was considered low when it did not exceed 20%. All works, except of Maurer et al., 2011 whom had a 22.7% lost in the placebo group met this limit (Table 2) [20].

**Table 1:** **Bias evaluation of included studies**

Study ID	Author	Domains					Reviewers observations
		Random sequence generation	Allocation Blinding	Participants and people blinding	Evaluators and outcome blinding	Incomplete outcome data	
		Method described with detail	Investigative personnel was blind to allocation	Blinding method for participants and personnel was effective?	Blinding method for the study evaluators was effective?	Main outcomes, fall outs, exclusions, number, in each intervention group, reason for losses and changes in plan analysis	
1	Maurer et al., 2011 [20]	Random sequence by validated system 1:1	Similar packages and presentation between placebo and intervention	Yes	Yes	Yes	-
2	Saini et al., 2011 [21]	Sequence 1:1:1:1	Similar packages and presentation between placebo and intervention	Yes	Yes	Yes	-
3	Maurer et al., 2013 [19]	Sequence 1:1:1:1 by voice interactive service	Similar packages and presentation between placebo and intervention	Yes	Yes	Yes	Blind in each center, drug not blinded but person administering the drug is blinded
4	Kaplan et al., 2013 [22]	3:1 stratified by itch severity score (ISS) and baseline weight	Similar packages and presentation between placebo and intervention	Yes	Yes	Yes	Person who prepared the drug was not blinded, but the one administering the drug was blinded
5	Saini et al., 2014 [23]	Sequence 1:1:1:1 stratified by weekly ISS, baseline weight (< or ?80 kg) and study center by interactive voice and web response system	Not specified in the article but the clinicaltrials.gov register specified the similar packages between placebo and intervention	Yes	Yes	Yes	Person who prepared the drug was not blinded but did not interact with patients

**Table 2:** **Inclusion and exclusion criteria, number of eligible randomized studies, sample size, interventions and patients lost from included studies**

Study	Author	Author inclusion criteria	Exclusion criteria	Eligible	Randomized	Sample Size	Interventions	Other treatment received	Treatment period	Follow-up period	Losses n(%)
1	Maurer et al., 2011 [20]	Moderade to severe CSU with persistent symptoms for ≥6 weeks in spite of treatment at maximum doses of antihistamines H1. UAS score >10 at the end of the test.	Acute urticaria, diarrhea, kidney failure, elevated IgE due to other allergy or urticaria reasons, epilepsy, antibiotic allergic reactions, malignancies in the last 5 years, CVA or ischemia, IV steroid use, methotrexate, cyclosporine or other immunosuppressant 4 weeks before	341	49	27	27 Omalizumab 75–375 mg dose according to weight, subcutaneous, once every 2 or 4 weeks for 24 weeks	H1 antihistamines, 10 mg of loratadine on demand and 1 mg of clemastine as rescue medication	24 weeks	**-**	2 (7.40)
						22	Placebo			**-**	5 (22.72)
2	Saini et al., 2011 [21]	Idiopathic chronic urticaria, for more than 3 months, no definitive cause, moderate to severe symptoms, pruritus and urticaria for more than 3 days in 7 days for a period of 6 weeks in spite of treatment with antiH1. UAS ≥4 or UAS7 ≥ 12 during run-in period before randomization.	Weight <40 kg, pregnancy or lactation, other skin disorder associated to pruritus, treatment with omalizumab 12 months before, contraindication for diphenhydramine, treatment with any other investigational drug 30 days before, clinically relevant disease that could affect the outcomes, impairment to complete follow up, use of immunosuppresants 3 months before hydroxicloroquine, methotrexate, sulfasalazine, dapsone, cyclophosphamide, intravenous immunoglobulin, plasmapheresis, other therapies with monoclonal antibodies, use of cyclosporine in the month before, use of antileukotriens or antihistamines H2 the week before.	119	90	23	Omalizumab 75 mg single dose sc	All patients were provided 25 mg of diphenhydramine to use as a rescue medication for pruritus relief on an as-needed basis. The maximum allowable daily dose of diphenhydramine was 75 mg in the United States and 50 mg in Germany. Patients who required any other medications(including systemic corticosteroids) to treat persistent/worsening diseasewere discontinued from the study	4 weeks	12 weeks	5 (21.73)
						25	Omalizumab 300 mg single dose sc			12 week	2 (8.0)
						21	Omalizumab 600 mg single dose sc			12 weeks	1 (4.76)
						21	Placebo1			12 weeks	1 (4.76)
3	Maurer et al., 2013 [19]	Idiopathic chronic urticaria for 6 months, presence of urticaria with pruritus for at least 8 consecutive weeks before inclusion in spite of consecutive use of antihistamines, UAS 7 ≥ 16 (range 0–42), weekly itch score < =8 (range 0–21) 7 days before randomization, without losses of electronic poll 7 days prior to randomization.	Cause of urticaria (physical), use of systemic glucocorticoids for 5 or more days, hydroxicloroquine, methotrexate, cyclosporine, cyclophosphamide, or intravenous immunoglobulin 30 days before. Use of antiH2 or leukotriens antagonists days before the visit (14 days prior randomization), use of antiH1 in higher doses than the allowed 3 days before the visit, previous history of cancer, weight < 20 kg, hypersensitivity to omalizumab, treatment with omalizumab in the previous year, pregnancy.	466	323	82	75 mg omalizumab, one injection every 4 weeks for 3 doses	Prerandomization H1-antihistamine throughout the treatment period. During the follow-up period, patients were permitted to use a licensed dose of one additional H1-antihistamine. For the duration of the study, all patients were provided with diphenhydramine (25 mg) as rescue medication for itch relief (up to a maximum of three doses in 24 hours on the basis of local regulations).	12 weeks	16 weeks	7 (8.53)
						83	150 mg omalizumab, one injection every 4 weeks for 3 doses			16 weeks	9 (10.84)
						79	300 mg omalizumab, one injection every 4 weeks for 3 doses			16 weeks	12 (15.18)
						79	Placebo, similar presentation to drug			16 weeks	5 (6.32)
4	Kaplan et al., 2013 [22]	12 to 75 years old, 18–75 years in Germany, idiopathic chronic urticaria for 6 months or more, pruritus and hives for more than 6 weeks before inclusion in spite of antiH1, antiH2, antileukotriens or both, UAS7 ≥ 16 and an itchy index of 8 or more, 7 days before randomization, UAS at the clinic of 0 or more in one of the visits, treatment with antiH1, antiH2, antileukotriens or both regime, 3 consecutive days, 14 days before, patient desires to participate, signs informed consent, no loss of symptoms 3 days prior to randomization	Cause of urticaria (physical), daily systemic steroids doses more than 5 days, use of hydroxicloroquine, methotrexate, cyclosporine, cyclophosphamide, or intravenous immunoglobulin 30 days before, previous history of cancer, hypersensitivity to omalizumab in the previous year, evidence of parasitic infection, history of anaphylactic shock, pregnancy or lactation, potential pregnancy not accepting contraception	480	336	252	Omalizumab 300 mg every 4 weeks for doses	Maintain stable doses of their prerandomization combination therapy with H1-antihistamine treatment plus H2-antihistamines, LTRAs, or both. For the duration of the study, patients were provided with 25 mg of diphenhydramine as rescue medication for symptom relief (up to a maximum of 3 doses per 24-hour period or fewer depending on local regulations).	24 weeks	16 weeks	31 (12.3)
						84	Placebo in the same presentation and administration			16 weeks	21 (25.0)
5	Saini et al., 2014 [23]	12-75 years old, (18–75 years in Germany), with diagnosis of CSU ≥ 6 months with hives and itching ≥8 consecutive weeks despite of anti H1 treatment. Use of an approved dosage of an H1 antihistamine for ≥3 consecutive days, UAS ≥ 4 on one or more screening days, UAS7 ≥ 16 an itch component of UAS7 ≥ 8 during the 7 days before randomization, willing to complete symptom diary, no missing eDiary entries during the 7 days before randomization	Clearly defined underlying etiology for chronic urticaria (cold, presure, etc.), presence of disease with symptoms of urticaria or angioedema, including hereditary or acquired angioedema, routine dosis of systemic steroids, hydroxychloroquine, methotrexate, cyclosporine, cyclophosphamide, or intravenous immunoglobulin ≤30 days of day −14. use of H2-antihistamines or LTRA ≤ 7 days of day −14. use of H1 antihistamines at greater than the approved doses ≤3 days of day −14. history of malignancy, weight <20 kg, hypersensitivity to omalizumab, previous treatment with omalizumab within the previous year.	483	319	78	Omalizumab 75 mg every 4 weeks for 6 doses	Maintain stable doses of their prerandomization H1 antihistamine treatment. Weeks 13 to 24 patients were allowed to add one additional H1 antihistamine. Patients were permitted to take diphenhydramine 25 mg as needed for itch relief (up to a maximum of three doses per 24 hours, or less if required by local regulations)	24 weeks	16 weeks	11 (14.10)
						80	omalizumab 150 mg every 4 weeks for 6 doses			16 weeks	16 (20.0)
						81	omalizumab 300 mg every 4 weeks por 6 doses			16 weeks	8 (9.87)
						80	placebo			16 weeks	19 (23.75)

Five studies were included in the systematic review, with a total of 1117 patients with CIU/CSU. The weighted average age was 42.07 years, predominantly of female sex (73%). Most of participants were Caucasian (85.6%) taking into account the populations included in these studies (German and American) although Saini et al., 2013 included patients from Denmark, France, Germany, Italy, Poland, Spain, Turkey and United States (Table 3) [19]-[23].

**Table 3:** **Demographic characteristics and mean weight and IgE of patients in the included studies**

Study ID	Interventions	Age (mean ± SD)	Female sex n(%)	Caucasian n(%)	Weight (kg) mean ± SD	<80 kg n (%)	IgE (IU/mL) mean ± SD	IgE (IU/mL) median (range)
1	Omalizumab 75-375	39.1 ± 9.0	19 (70.4)	27 (100.0)	81.9 ± 20.2	-	211 ± 158	-
	Placebo	42.3 ± 15.0	19 (86.4)	22 (100.0)	71.2 ± 12.4	-	181 ± 136	-
2	Omalizumab 75	38.8 ± 15.5	15 (65.2)	20 (87.0)	80.5 ± 21.6	14 (60.9)	251.5 ± 389.6	62 (3–1500)
	Omalizumab 300	42.9 ± 15.7	17 (68.0)	19 (76.0)	82.2 ± 22.8	13 (52.0)	170.5 ± 178.5	131.5 (2–819)
	Omalizumab 600	40 ± 11.1	12 (57.1)	18 (85.7)	80.6 ± 18.1	11 (52.4)	134.9 ± 142.9	90 (4–617)
	Placebo	41.2 ± 16.2	17 (81)	18 (85.7)	80.4 ± 24.8	13 (61.9)	297.4 ± 748.9	62 (3–1500)
3	Omalizumab 75	39.7 ± 15.0	61 (74.0)	64 (78.0)	82.8 ± 21.2	43 (52.4)	168.2 ± 321.9**	79
	Omalizumab 150	43.0 ± 13.2	65 (79.0)	70 (85.0)	82.4 ± 20.7	41 (49.4)	-	-
	Omalizumab 300	44.3 ± 13.7	63 (80.0)	68 (86.0)	80.3 ± 19.9	41 (51.9)	-	-
	Placebo	43.1 ± 12.5	55 (70.0)	70 (89. 0)	84.3 ± 25.7	41 (51.9)	-	-
4	Omalizumab 300	42.7 ± 17.9	186 (73.8)	223 (88.5)	29.4 ± 7.1*	-	162.3 ± 306.4	79 (1–3050)
	Placebo	44.3 ± 14.7	55 (66.3)	75 (90.4)	31 ± 9.6*	-	147.2 ± 224.4	71 (1–1230)
5	Omalizumab 75	40.7 ± 15.2	55 (71.4)	62 (80.5)	81.1 ± 19.2	38 (48.7)	-	91 (1–2030)
	Omalizumab 150	41.1 ± 14	64 (80.0)	63 (78.8)	83.2 ± 24.4	40 (50.0)	-	71 (1–5000)
	Omalizumab 300	42.4 ± 13.2	60 (74.1)	74 (91.4)	81.6 ± 19.7	45 (55.5)	-	85.5 (1–2330)
	Placebo	40.4 ± 15.6	52 (65.0)	64 (80.0)	83 ± 20.5	35 (43.7)	-	92 (1–1010)

*BMI (kg/m2). **mean and SD of all patients included. SD: standard deviation. IgE: Immunoglobulin E normal range 13 to 127 IU/mL.

Some studies had more than one intervention, comparing different doses of omalizumab vs. placebo every 2 or 4 weeks (75 mg, 150 mg, 300 mg or 600 mg) and different follow-up periods (4–24 weeks). In one study, the dose of omalizumab was calculated according to the weight and the levels of immunoglobulin E (ranging between 75 and 375 mg) [20]. In Table 2 we describe the study, inclusion criteria, exclusion criteria, sample size, interventions, number of participants included in the intervention and placebo group, time of follow-up and patients lost to follow up or abandonment of treatment.

### Effectiveness

The main outcomes were the change in UAS7 in different periods of follow-up, change in the ISS in the last 7 days from baseline, proportion of patients with UAS7 less or equal to 6 in follow-up periods, change in daily score of urticaria during follow up as well as change in the size score of urticaria (wheal). Outcomes such as the improvement in DLQI and Cu-Q2oL were also included. Safety outcomes such as frequency of at least one adverse event and frequency of adverse events suspected to be caused by the drug were included (Tables 4, 5 and 6).

**Table 4:** **Description of urticaria activity score, weekly itch severity score, and the magnitude of change before and after treatment**

Study ID	Sample size	Intervention	UAS7 baseline (mean ± SD)	Change in UAS7 (mean ± SD)	UAS < 6 n (%)	ISS baseline (mean ± SD)	Change in weekly ISS (mean ± SD
1	27	Omalizumab 75-375 mg	24.6 ± 7.4	−17.8^Ϯ^	-	-	-
	22	Placebo	21.3 ± 7.6	−7.9	-	-	-
2	23	Omalizumab 75 mg	27.3 ± 8.31	−9.8 ± 11.75	-	13.1 ± 3.53	−4.5 ± 5.84
	25	Omalizumab 300 mg	27.3 ± 7.19	−19.9 ± 12.38^Ϯ^	-	13 ± 3.72	−9.2 ± 5.98^Ϯ^
	21	Omalizumab 600 mg	26.8 ± 6.98	−14.6 ± 10.17	-	12.6 ± 3.19	−6.5 ± 5.63
	21	Placebo	31 ± 7.32	−69 ± 9.84	-	14 ± 4.23	-3.5±5.22
3	82	75 mg omalizumab	30.7 ± 6.9	-	22 (27)	14 ± 3.7	-5.9±6.5
	83	150 mg omalizumab	31.4 ± 7.0	-	35 (43)^Ϯ^	14.2 ± 4.1	-8.1±6.4^Ϯ^
	79	300 mg omalizumab	29.5 ± 6.9	-	52 (66)^Ϯ^	13.7 ± 3.5	-9.8±6.0^Ϯ^
	79	Placebo	31 ± 6.6	-	15 (19)	14 ± 3.4	-5.1±5.6
4	252	Omalizumab 300 mg	31.2 ± 6.6	−19 (20.6 a-17.4)*^Ϯ^	132 (52)^Ϯ^	14 ± 3.6	-8.6 (-9.3 a -7.8)*^Ϯ^
	84	Placebo	30.2 ± 6.7	−8.5(−11.1 a-5.9)*^Ϯ^	10 (12)	13.8 ± 3.6	-4.0 (-5.3 a -2.7)*
5	78	Omalizumab 75 mg	31.7 ± 6.7	−13.82 ± 13.26^Ϯ^	20 (26)	14.5 ± 3.6	-6.46±6.14^Ϯ^
	80	Omalizumab 150 mg	30.3 ± 7.3	−14.44 ± 12.95^Ϯ^	32 (40)	14.1 ± 3.8	-6.66±6.28^Ϯ^
	81	Omalizumab 300 mg	31.3 ± 5.8	−20.75 ± 12.17^Ϯ^	42 (52)	14.2 ± 3.3	-9.4±5.73^Ϯ^
	80	Placebo	31.1 ± 6.7	−8.01 ± 11.47	9 (11)	14.4 ± 3.5	-3.63±5.22

UAS7: Urticaria Activity Index in 7 days, ISS: Itch Severity Score, SD: standard deviation, *mean and 95% confidence interval, ^Ϯ^ p<0.01 compared to placebo.

**Table 5:** **Description weekly urticarial score, DLQI, Cu-Q2oL, presence of angioedema at baseline and change by intervention administered**

Study ID	Sample size	Intervention	Weekly urticaria score at baseline	Change in weekly urticaria score (mean ± SD)	DLQI baseline (mean ± SD)	Change in DLQI (mean ± SD)	Cu-Q2oL improvement (%)	Presence of angioedema baseline, n (%)	Angioedema free days (%)
1	27	Omalizumab 75-375 mg	-	−9,2^Ϯ^	-	62,4*	50	-	77,8
	22	Placebo	-	−3,3	-	15,3*	6,3	-	36,4
2	23	Omalizumab 75 mg	14,2 ± 5,71	−5,3 ± 6,91	-	-	-	-	-
	25	Omalizumab 300 mg	14,7 ± 4,62	−10,7 ± 6,75^Ϯ^	-	-	-	-	-
	21	Omalizumab 600 mg	14,2 ± 4,81	−8,1 ± 6,0	-	-	-	-	-
	21	Placebo	17 ± 4,79	−3,5 ± 5,17	-	-	-	-	-
3	82	75 mg omalizumab	16,8 ± 4,2	−7,2 ± 7,0	12,6 ± 6,5	−7,5 ± 7,2	-	31 (38)	93,5
	83	150 mg omalizumab	17,1 ± 4,1	−9,8 ± 7,3^Ϯ^	13 ± 6,1	−8,3 ± 6,3	-	38 (46)	91,6
	79	300 mg omalizumab	15,8 ± 4,6	−12,0 ± 7,6^Ϯ^	12,7 ± 6,4	−10,2 ± 6,8^Ϯ^	-	32 (41)	95,5
	79	placebo	17 ± 4,2	−5,2 ± 6,6	12,6 ± 5,9	−6,1 ± 7,5	-	30 (38)	89,2^Ϯ^
4	252	Omalizumab 300 mg	17,1 ± 4,2	−10,5 (−11,4 a −9,5)^*Ϯ^	-	−9,7 (−10,6 a −8,8)^*Ϯ^	−3,9 (−4,9 a −3,0)^*Ϯ^	137 (54,4)	91^Ϯ^
	94	Placebo	16,4 ± 4,6	−4,5 (−5,9 a −3,1)^*^	-	−5,1 (−7,0 a −3,2)^*^	−2,7 (−3,8 a −1,6)^*^	41 (49,4)	88,1
5	78	Omalizumab 75 mg	17,2 ± 4,2		12,8 ± 6,1			35 (45,5)	
	80	Omalizumab 150 mg	16,2 ± 4,6		13,6 ± 7,1			38 (47,5)	
	81	Omalizumab 300 mg	17,1 ± 3,8		13,0 ± 6,7	10,29 (7,3)		34 (42,0)	96,1
	80	Placebo	16,7 ± 4,4		14,0 ± 6,6	6,13 (5,25)		44 (55,0)	88,2

SD: standard deviation, DLQI: Dermatologic Life Quality Intex, Cu-Q2oL: Chronic urticaria Quality of Life Questionnaire, ^*^mean and 95% confidence interval, ^Ϯ^ p < 0.01 compared to placebo.

**Table 6:** **Adverse events reported in the included studies**

Study ID	Sample size	Intervention	At least 1 adverse event n(%)	Adverse event during follow up, n(%)	Event allegedly caused drug n(%)	Serious adverse event n(%)
1	27	Omalizumab 75-375 mg	22 (81.5)	-	6 (22.2)	-
	22	Placebo	19 (86.4)	-	6 (22.7)	-
2	23	Omalizumab 75 mg	8 (34.8)	9 (50.0)	-	-
	25	Omalizumab 300 mg	12 (48.0)	12 (52.2)	-	-
	21	Omalizumab 600 mg	10 (47.6)	5 (25.0)	-	-
	21	Placebo	10 (47.6)	7 (35.0)	-	-
3	82	75 mg omalizumab	45 (59)	-	7 (9)	4 (5)
	83	150 mg omalizumab	59 (67)	-	8 (9)	5 (6)
	79	300 mg omalizumab	51 (65)	-	7 (9)	6 (8)
	79	placebo	48 (61)	-	3 (4)	7 (9)
4	252	Omalizumab 300 mg	211 (83.7)	-	28 (11.1)	18 (7.1)
	84	Placebo	65 (78.3)	-	11 (13.3)	5 (6)
5	78	Omalizumab 75 mg	55 (78.6)	36 (51.4)	6 (8.6)	2 (2.9)
	80	Omalizumab 150 mg	72 (82.8)	45 (51.7)	9 (10.3)	5 (5.7)
	81	Omalizumab 300 mg	57 (70.4)	38 (46.9)	14 (17.3)	2 (2.5)
	80	Placebo	53 (66.3)	32 (40.0)	4 (5.0)	5 (6.3)

Maurer et al., reported a significant reduction in UAS7 using omalizumab (75-375 mg every 2 to 4 weeks for 24 weeks) compared with placebo at 24 weeks follow-up (change −17.8 UAS7 in omalizumab , −7.9 in placebo p = 0.0089) [20]. They reported a smaller area under the curve for UAS in the omalizumab group, compared with placebo (p = 0.0002). They also reported a reduction in urticarial lesions score (−9.2 vs. -3.3, respectively, p = 0.0019) and absence of angioedema in 77.8% of patients, compared with 36.4% in the placebo group [20].

Saini et al., compared the impact of single doses of omalizumab of 75 mg, 300 mg and 600 mg vs. placebo, on UAS7 at 4 weeks, with an additional 12 weeks of follow-up to monitor security [21]. They reported significant differences in the dose of 300 mg and 600 mg compared with placebo (300 mg −19,9 vs. -6,9 p < 0,01; 600 mg −14,6 vs. -6,9 p = 0,047). There were also differences in the ISS at week 4 of follow-up between the dose of 300 mg of omalizumab vs. placebo (−9.2 ± 5.98 vs. -3.5 ± 5.22 p <0.001), but not with 75 mg or 600 mg (75 mg −4.5 ± 5.84, p 0.16, 600 mg −6.5 ± 5.63 p = 0.56). Regarding the number of weekly urticarial lesions, a single dose of 300 mg omalizumab lowered this score on average 10.7 ± 6.75, which is significant compared to placebo (−3.5 ± 5.17 p <0.001). The dose of 600 mg also significantly lowered this score (600 mg −8,1 ± 6,0 p = 0,02) [21].

Maurer et al., in 2013 reported a randomized double blind clinical trial comparing the use of 75 mg, 150 mg and 300 mg of omalizumab vs. placebo every 4 weeks for 3 months, followed by 16 weeks of observation [19]. The primary outcome was change in ISS at week 12 compared to baseline, showing significant reductions in mean ISS with doses of 150 mg and 300 mg compared to placebo (150 mg −8.1 ± 6.4 300 mg −9.8 ± 6.0, placebo −5.1 ± 5.6 p < 0.01 y <0.001 respectively). Similar behavior was also observed in the number of urticarial lesions at week 12, the percentage of patients with UAS7 ≤ 6, and the rate of dermatology life quality at week 12, but not with the dose of 75 mg (Tables 4 and 5). There was also a higher rate of angioedema-free days in the group receiving 300 mg of omalizumab compared to placebo (95.5% vs. 89.2% p <0.001), a difference that was not observed with doses of 150 or 75 mg (91.6% and 93.5% respectively) [19].

Kaplan et al. in 2013, compared the dose of 300 mg of omalizumab every 4 weeks for 6 months vs. placebo in the treatment of CIU/CSU, including other treatment options such as H2 antihistamines and leukotriene modifiers [22]. They found a significant change at week 12 in the ISS (−8.6 95% CI −9.3 to −7.8 vs. -4.0 95% CI −5.3 to −2.7 respectively, p <0.001), in the UAS7 (−19 95% CI −20.6 to −17.4 vs. -8.5, 95% CI −11.1 to −5.9 p <0.001), in the weekly score of urticarial lesions (− 10.5 95% CI −11.4 to −9.5 vs. -4.5 95% CI −5.9 to −3.1 p <0.001) in the DLQI (−9.7 95% CI −10.6 to −8.8 vs. -5.1 95% CI −7.0 to −3.2 p <0.001), and the percentage of days free of angioedema (95% CI 91 88 2 to 93.8 vs. 88.1 95% CI 83.6 to 92.7 p <0.001) (Tables 4 and 5) [22].

Saini et al. in 2014, compared the dose 75 mg, 150 mg, and 300 mg every 4 weeks for 6 months vs placebo in patients with CIU/CSU, despite the use of H1-antihistamine. They reported a statistical improvement in UAS7 (mean change −13.82 in 75 mg, −14.4 in 150 mg, −20.75 in 300 mg vs −8.01 in placebo), ISS (mean change −6.46 in 75 mg, −6.66 in 150 mg, −9.4 in 300 mg vs 3.63 in placebo) and angioedema free days 96.1% in 300 mg vs 88.2% in placebo (Tables 4 and 5) [23].

After the end of treatment period, three studies reported the recurrence of symptoms in terms of ISS and weekly score of hives. Maurer et al. 2013 reported statistical significant differences between 300 mg of omalizumab and placebo at week 18 (six weeks after ending the treatment) being similar to placebo after 18 week. Also, 300 mg of omalizumab reduced the weekly score of hives at week 18 comparted to placebo [19]. Kaplan et al. 2013 reported during the follow-up period significant differences between omalizumab 300 mg and placebo at 33 week in the ISS score. These gradually increased to values similar to those in the placebo group after the 33 week, there were no statistical differences between omalizumab and placebo groups at week 40 [22]. Saini et al. 2014 reported after 24 week, the ISS difference of omalizumab 300 mg compared to placebo maintained to the 31 week, the ISS increased to values similar to those in the placebo group and at the end of the follow up there were no differences between omalizumab and placebo groups [23].

There are also multiple case series and uncontrolled studies reporting the benefit of this drug in the treatment of chronic idiopathic urticaria (Table 7).

**Table 7:** **Summary of non-controlled studies and outcomes**

Effects of omalizumab in patients with urticaria: uncontrolled studies					
Author (year)	Urticary type	N	No response	Partial response	Complete response
Ivyanskiy (2012)	CIU 12, AIU 6, DPU 1	19	3	5	11
Ferrer (2011)	CSU	9	2	5	2
Groffik (2011)	CSU	9	0	4	5
Sánchez-Machín (2011)	CSU	1	0	0	1
Saavedra (2011)	CSU	1	0	0	1
Krause (2010)	Dermographic U	1	0	0	1
Buller Kotte (2010)	Heat U	1	0	0	1
Binslej-Jensen(2010)	DPU	1	0	0	1
Magerl (2010)	CSU	8	0	1	7
Al-Ahmad (2010)	AIU	3	0	0	3
Kemoli (2010)	AIU	1	0	0	1
Sabroe (2010)	Cholinergic U	1	1	0	0
Waiber (2009)	Solar U	1	0	1	0
Maspero (2009)	AIU	1	0	0	1
Kaplan (2008)	AIU	12	1	4	7
Güzelbey (2008)	Solar U	1	0	0	1
Metz (2008)	Cholinergic U	1	0	0	1
Godse (2008)	CSU	3	0	0	3
Sands (2007)	CAU	3	0	0	3
Spector (2007)	2 AIU, 1 CSU	3	0	0	3
TOTAL		78	7(8,9%)	20 (25,6%)	51 (65,3%)

CIU: Chronic Idiopathic Urticaria; AIU: Aspirin induced Urticaria; DPU: Delayed Pressure Urticaria; CSU: Chronic Spontaneous Urticaria; CAU: Chronic Autoinmune Urticaria.

### Safety

Maurer et al. in 2011, reported a similar incidence of adverse events suspected to be caused by omalizumab and placebo (22.2% and 22.7% respectively), with no clinical evidence or any trends in laboratory parameters that were associated with treatment with omalizumab [20]. They reported 44% of adverse events during the treatment period (day 0 to week 4) (placebo 47.6%, omalizumab 75 mg 34.8%, 300 mg 48% and 600 mg 47.6%) [21]. Adverse events requiring priority treatment greater than 5% were: upper respiratory tract infection, headache, nasopharyngitis, and dysmenorrhea. During follow-up (week 4–16), 40.7% of patients experienced at least one adverse event (35% placebo, 75 mg omalizumab: 50%, 300 mg: 52.2%, 600 mg: 25%). 4.4% of participants had an adverse event that led to discontinuation of treatment (pregnancy, asthma, itching -Dose 75 mg-, exacerbation of urticaria -Dose 600 mg-) [20].

Maurer et al. in 2013 reported 59%, 67% and 65% of adverse events at doses of 75, 150 and 300 mg of omalizumab, respectively, compared to 61% in the placebo group [19]. Nine serious adverse events were reported, of which 5 were in the group receiving 300 mg of omalizumab, two in the placebo group, one in the group receiving 75 mg and one in the group receiving 150 mg. Most events were reported during study phases where patients were not receiving active treatment.

Kaplan et al. reported an 83.7% frequency of one or more adverse events in the group receiving 300 mg of omalizumab for 16 weeks and 78.3% of adverse events in the placebo group [22]. Of these, a relation with the intervention was suspected in 11.1% and 13.5% respectively, and 7.1% and 6.0% were considered serious adverse events respectively. Adverse events requiring priority treatment were mainly gastrointestinal effects (nausea, diarrhea, abdominal pain), and presented at similar frequency in both groups. During the treatment period, 65.1% of adverse events occurred in omalizumab-treated patients vs. 63.9% in the placebo group, the most common being headache and upper respiratory tract infections in the omalizumab group and nasal congestion, migraine and idiopathic urticaria in the placebo group. In the follow-up period, the incidence of adverse events was similar in both groups (52% vs. 47% respectively). There was 2.8% of serious adverse events with omalizumab and 3.6% with placebo. No serious adverse events related to treatment were reported in this study.

Saini et al. 2014 reported any adverse event in 300 mg of omalizumab 70.4%, 82.8% in 150 mg, 78.6% in 75 mg and 66.3% in the placebo group. The most common symptoms were headaches, arthralgia and injection-site reactions in omalizumab group as compared with placebo. The proportion of patients with adverse events reported as suspected to be cause by the study drug increased as the dose of omalizumab increased. There were 2 severe events in 150 mg and 300 mg groups. In this study three patients had suspected anaphylaxis, two of them during omalizumab treatment and one 142 days post final dose of study drug althought the anaphylaxis was not attributed to study drug [23].

## Discussion

Although CIU/CSU is a disease with a low probability of death, it has a high impact on the quality of life of affected individuals. The therapeutic options available, mainly H1 antihistamines and anti-leukotrienes may not be sufficient to achieve adequate control of symptoms [8]. In this subgroup of patients in whom the use of immunosuppressive agents is indicated as an alternative, the use of omalizumab for the treatment of CIU/CSU has been proposed [3],[8],[18]. Although little information is yet available about this drug, its safety has been demonstrated in patients with asthma. This is the first systematic review of omalizumab in chronic idiopathic urticaria reported in the literature. Of 1117 patients obtained, 831 received at least one dose of omalizumab in randomized controlled clinical trials with placebo. There are also multiple case series and uncontrolled studies reporting the benefit of this drug in the treatment of various types of CU.

The different doses used in the studies show a clear benefit of using omalizumab 300 mg compared with placebo in the treatment of the disease. One of the most important limitations was the use of different doses across studies, time of management, and follow-up of results, which ranged from single dose to monthly doses for 24 weeks. However, three studies evaluated the dose of 300 mg that resulted in improvement of UAS7, ISS and urticaria score compared with placebo. Another important limitation was the quantitative score of change in the different scales, from a categorical scale of severity of symptoms, making it difficult to perform a clinical interpretation of the reduction in the average of each of the scales. However, the authors reported a significant minimum difference, and also on these scales, the score of 0 means no symptoms and the maximum score a greater intensity of symptoms. The main strength of the studies was that they were randomized clinical trials with adequate methodology and low loss of patients during follow-up.

With the above, although no meta-analysis was performed by differences in the dose and times of treatment, it can be concluded that the dose of 300 mg of omalizumab appears to be effective in treating CIU/CSU, but it is associated with a higher frequency of adverse effects (headache and upper respiratory infection). Further studies evaluating the efficacy of the dose of 300 mg of omalizumab in different population groups are needed, since the reported studies included patients from Germany and the United States mainly. The duration of effective treatment with fewer incidence of adverse events must also be determined.

## Authors’ information

DCC: Specialist in Internal Medicine and Epidemiology. Fundación Valle del Lili. Cali, Colombia EG: Specialist in Allergy and Clinical Immunology. Chief of Allergy Unit. Fundación Santa Fé de Bogotá. Bogotá, Colombia EE: Specialist in Internal Medicine and Allergology. Proffesor Universidad del Norte. Barranquilla, Colombia. MSB: Specialist in Allergy and Clinical Immunology. Centro Médico-Docente La Trinidad. Caracas, Venezuela. CDS: Specialist in Internal Medicine and Allergology. Chief of Allergy Unit. Fundación Valle del Lili. Cali, Colombia

## Authors’ original submitted files for images

Below are the links to the authors’ original submitted files for images. Authors’ original file for figure 1
