# Supplementary figures and images for: Omalizumab vs. placebo in the management of chronic idiopathic urticaria: a systematic review
Source: World Allergy Organ J. 2014 Dec 31;7(1):72. doi: 10.1186/s40413-014-0050-z (PMC4280746; doi:10.1186/s40413-014-0050-z)

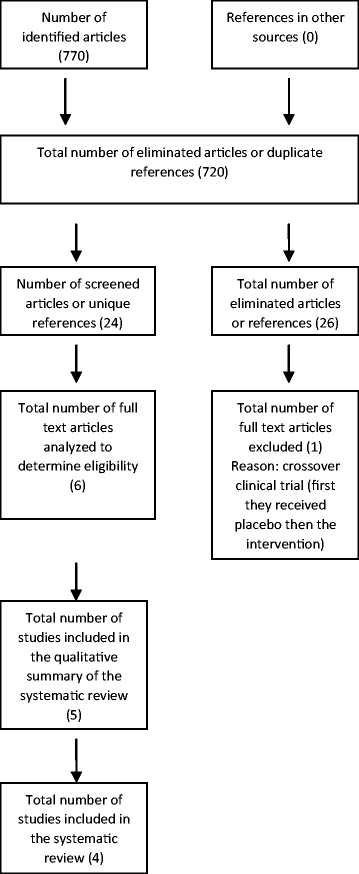

Supplement: Supplementary file 1 — Authors’ original file for figure 1 [file 40413_2014_50_MOESM1_ESM.gif]
